# Microbiome-Mediated Cd Stabilization in Chilli Pepper: Roles of Capsaicinoids and Cultivar Genetics Under Environmental Stress

**DOI:** 10.3390/plants15040630

**Published:** 2026-02-16

**Authors:** Irfan Haidri, Qudrat Ullah, Muhammad Qasim, Muhammad Ali Amir, Waqas Haider, Hien Huu Nguyen, Athakorn Promwee

**Affiliations:** 1 School of Agricultural Technology and Food Industry, Walailak University, Nakhon Si Thammarat 80160, Thailand; irfan.ha@mail.wu.ac.th (I.H.); qudratullahmpur@gmail.com (Q.U.); muhammadqasimgcuf@gmail.com (M.Q.); muhammad.al@mail.wu.ac.th (M.A.A.); waqashaidar01@gmail.com (W.H.); 2 School of Agriculture and Natural Resources, Vinh University, Vinh City 43108, Vietnam; hiennh@vinhuni.edu.vn; 3 Herbology Research Center, Walailak University, Nakhon Si Thammarat 80160, Thailand

**Keywords:** *Capsicum annuum*, cadmium, capsaicinoids, phytostabilisation, rhizobiome, Pun1 locus, volatilome, rhizosphere engineering

## Abstract

Chilli pepper agroecosystems (*Capsicum annuum* L.) are increasingly threatened by cadmium (Cd) contamination, with emerging climatic stressors such as drought further exacerbating risks to food safety and crop productivity. This review synthesizes current evidence on microbiome-mediated Cd phytostabilisation in chilli pepper, with a particular focus on the roles of capsaicinoids and cultivar-specific genetic regulation in shaping rhizosphere microbial communities. Existing studies demonstrate that capsaicinoid-rich cultivars selectively recruit specialized rhizosphere microbes, enhancing root-level Cd sequestration and achieving Cd retention efficiencies of approximately 40–55%, thereby substantially restricting Cd translocation to edible fruit tissues. Multi-strain plant growth-promoting rhizobacteria (PGPR) consortia, especially when combined with structured organic amendments, have been reported to reduce fruit Cd and nickel (Ni) accumulation by more than 87% in contaminated soils. These responses are regulated by pungency-associated genetic loci, including Pun1 (pungency locus 1) and Pun4 (pungency locus 4) genes, which influence secondary metabolism and microbial assembly under metal stress conditions. The review highlights key knowledge gaps regarding the long-term stability of engineered rhizobiomes, the in situ dynamics of the Capsicum volatilome as a microbial recruitment signal, and the interactive effects of Cd contamination and drought in field environments. Overall, this synthesis provides a mechanistic framework for deploying high-pungency cultivars and microbiome-based strategies to improve Cd phytostabilisation, with important implications for sustainable chilli production in drought-prone, metal-contaminated agroecosystems.

## 1. Introduction

Chilli pepper is a globally important horticultural crop valued for its culinary uses and high nutritional content, including vitamins C and A, carotenoids, fiber, and phenolic compounds. It is cultivated across tropical and temperate regions, with major production in South and East Asia and the Americas, but its productivity and food safety are increasingly threatened by heavy metal contamination and climatic instability [1]. Chilli peppers are the oldest crops grown in the Colombian region and have been an important part of human diets and the global spice trade [2]. Recent studies in specific production areas, such as Bangladesh and India, highlight the crop’s economic value. Indigenous Bangladeshi cultivars, including Naga, Dhani, and Kajini, are valued for their unique and rich bioactive substance components. In industrialised regions like the Shazand plain, severe pollution load indices (PLI) reaching 71.17 in irrigated fields underscore the high-risk environments where these high-value crops are cultivated [3].

Cd pollution has become a worldwide environmental issue, which threatens both food safety and crop yield. The main sources of Cd in intensive agricultural areas are the excessive use of phosphatic fertilisers, the use of sewage sludge, manure, and atmospheric pollutant deposition [4,5]. The Cd levels in the typical agricultural soils have been reported to be between 0.14 and 0.45 mg kg^−1^, with the bioavailability of the element being highly dependent on the soil pH; acidic soils are much more phytoavailable than alkaline soils [6]. Stresses are caused by climate change due to severe droughts and the increased toxicity of heavy metals in chilli agroecosystems. Drought stress causes a severe effect on the water status of plants, and the relative water content (RWC) decreases by about 30% in leaf and root tissues after 14 days of irrigation suspension [7]. Also, unpredictable climatic conditions and intensive agricultural activities change the water cycle in the soil, which may enhance salinity and lead to the leaching of contaminants into the groundwater aquifers, where heavy metals like Cr have been detected at levels of 19 µg L^−1^ [3,8].

Although capsaicinoids are best known for giving *Capsicum* fruits their characteristic heat, these features are unravelled by recent studies for their multifunctional properties. These compounds can act as signalling molecules within the plant and can influence the composition and activity of the rhizobiome [9]. The biosynthesis of capsaicinoids involves the combination of products from the phenylpropanoid and branched-chain fatty acid pathways. Genomic regulation mainly involves the Pun1 locus (AT3 and CS), which encodes capsaicin synthase [10]. Telomere-to-telomere (T2T) gapless genome assemblies have identified 117 potential capsaicinoid biosynthesis genes (CBGs), with CS-2, MYB31, and MYB48 specifically expressed in the placenta [11]. More regulators, such as the Pun4 locus (candidate gene DEMF06G16460 encoding 3-ketoacyl-CoA synthase), further influence the degree of pungency and metabolic flow [12].

Capsaicinoids and their precursors are not limited to fruit tissues but are part of the metabolic reaction of the plant to external environmental stress. Studies have found that high organic carbon and microbial activity in certain types of soils, like alluvial soil, have been known to promote higher levels of capsaicin accumulation and synthase activity compared to the lateritic soils [13]. These secondary metabolites, and a complex volatilome of more than 2734 compounds, are released to the rhizosphere, which are important chemical signals that mediate ecological interactions between plants and soil microbes [14]. Some recent studies found that capsaicin sends a selective signal in microbial recruitment. It affects the microbiome composition by promoting a beneficial microbial community and decreasing harmful pathogenic groups [9]. These metabolites regulate the assembly of rich bacterial phyla, including *Pseudomonadota* and *Bacteroidota*, which constitute 91.05% of the root-associated community in the rhizosphere [15]. The metabolites help to make a better community of plant growth-promoting rhizobacteria (PGPR), which are essential in increasing plant resilience to Cd and drought stress.

Depending on the intensity of metabolism, different cultivars have varying responses to Cd and drought stress. The strongest response to water deficit is observed in high-pungency genotypes, including ‘Jolokia’ (SHU 391,000–1,000,000), which undergoes the strongest reaction to water deficit by increasing capsaicinoid synthesis [16]. On the other hand, less pungent varieties, including Shishito, are regulated by quantitative trait loci (QTLs) that lead to high variation in capsaicinoid reduction (39.8% in Shql3 and 19.7% in Shql7). This process affects their ability to utilise pungency-related defence mechanisms in drought and heavy metal stress [17]. The accumulation of toxic metals in the edible portions of chilli peppers is very dangerous to human health. The recent studies show that fruit Cd levels may go up to 0.345 mg kg^−1^ with high Cd stress, which often exceeds international safety levels, including the 0.05 mg kg^−1^ wet weight standard established by the European Commission (EC Regulation No. 1881/2006) [6,15]. Children living in polluted areas have higher values of Estimated Daily Intake (EDI) compared to adults, and there is an urgent need to develop strategies and mechanisms that reduce root-to-fruit metal translocation [18]. The capsaicinoids are biosynthesised by joining the phenylpropanoid pathway, which furnishes the vanillyl moiety of phenylalanine, and the branched-chain fatty acid pathway, which furnishes the branched acyl moiety of valine or leucine. Capsaicin synthase (catalysed by Pun1 locus AT3 and CS genes) is the last step in the condensation process and is mainly expressed in the placenta of fruits. The flux of the pathway is controlled by regulatory factors, including transcriptional factors MYB31 and MYB48, and the Pun4 locus, which encodes 3-ketoacyl-CoA synthase.

Recent review studies have examined cadmium phytostabilisation mechanisms or Capsicum-associated rhizobiomes; however, these processes are most often discussed in isolation. Existing syntheses typically focus either on plant-based metal immobilisation strategies or on microbial community responses to environmental stress, with limited consideration of secondary metabolism as a coordinating regulatory factor. In this context, the present review advances an integrated framework that links capsaicinoid biosynthesis, cultivar-specific genetic regulation, and rhizosphere microbial assembly into a unified capsaicinoid rhizobiome Cd stabilisation axis. By integrating evidence from molecular genetics, plant economic strategies, volatilome-mediated microbial interactions, and functional microbial traits under drought and metal stress, this framework illustrates how pungency gradients are associated with the formation of stable rhizobiomes that enhance root Cd sequestration and restrict metal transfer to fruits, providing a mechanistic basis for climate-resilient chilli production in contaminated soils.

The future of research needs to focus on an interdisciplinary approach that combines genome regulation, management of microbiomes, and targeted metabolic profiling. The review aims to conclude the existing studies of Cd uptake behavior and the molecular mechanisms that regulate root uptake and translocation of fruits in chilli plants, and the processes of capsaicin-modulated rhizobiome assembly. It also aims to identify the role of capsaicin in shaping the rhizobiome. By integrating these processes, we propose a research framework to develop climate-resilient *Capsicum* cultivars that can effectively decrease and stabilise Cd.

## 2. Cd Dynamics in Chilli Agroecosystems

The accumulation of Cd into soil, which is mainly caused by the overuse of phosphatic fertilisers, sewage sludge, manure, and atmospheric deposition, is a major threat to chilli agroecosystems’ stability [4]. As Cd is a highly toxic trace element, it disturbs and affects the physiological and molecular systems of Chilli plants. Such phenomena highlight the need for thorough knowledge of its chemical behaviour and bioavailability in the soil–plant–water ecosystem.

### 2.1. Chemistry of Soil and Metal Speciation at Varying Moisture

The Cd mobility in soil is determined by the interactions of organic and inorganic components. In the system ecosystem, the Cd competes with macronutrients (N, P, K, Ca, Mg, S) and micronutrients (Fe, Zn, Cu, Ni) to occupy root absorption sites [19]. These interactions are strongly altered by soil physicochemical characteristics, such as pH, redox potential, and organic matter content. As an example, in rhizosphere soil, a major reduction in exchangeable metal fractions (in particular, Hg) is commonly accompanied by an increase in organically bound fractions. These shifts indicate the contribution of organic matter in metal transformation with in rhizosphere [4,20]. Alluvial soils with high water holding capacity (WHC) and nutrient-rich status have been found to promote various metabolic processes compared to the lateritic soils [21].

The bioavailability of heavy metals is closely associated with particular geochemical fractions in soil. In heavy metal contaminated soil, Cd has a significant portion of 59.7% is usually interlinked with Fe and Mn oxide-bound fractions. Additionally, the main sources of uptake of Cd by plants form the water-soluble and exchangeable pools [22]. The isotopic analysis of Cd fractionation suggested that the soil–plant system is closely associated with drought resistance. In vegetables with low drought resistance, such as green pepper, preferential transport through the xylem results in little to no, or even negative, Cd isotope fractionation between straw and fruit (Δ^114^/^110^Cd ranging from 0.01 ± 0.04‰ to −0.34 ± 0.02‰). In contrast, drought-tolerant species show positive isotope fractionation [23]. These findings indicate that the size of the bioavailable Cd pool, as well as its sequestration in roots, can be altered by plant-driven processes, particularly the release of root exudates such as organic acids.

Soil pH and the application of organic amendments serve as critical regulators of metal solubility. The introduction of livestock manure can either promote or inhibit metal accumulation. For example, pig and cattle manure have been found to reduce metal activity by increasing organic acid secretion and decreasing soil redox potential [24]. This process facilitates the reduction of metals to more stable forms, such as the reduction of Tl^3+^ to Tl^+^, thereby decreasing the exchangeable fraction [25]. In chilli cultivars, Cd stress triggers a dose-dependent decline in growth and biochemical attributes. At a concentration of 5 mg Cd kg^−1^ soil, varieties such as Desi (V2) and G-916 (V4) exhibited substantial decreases in carotenoid levels (−197.39% and −138.78%, respectively), whereas intermediate varieties such as Sathra (V3) showed a comparatively smaller reduction (−10.37%). The antioxidant defence system also responds to these shifts; maximum decreases in peroxidase (POD), superoxide dismutase (SOD), and catalase (CAT) activities were recorded at 5 mg kg^−1^ Cd stress, reaching −31.81%, −25.98%, and −16.39%, respectively, in the F1-9226 variety [4]. Additionally, the higher Cd concentrations (2 mM and 4 mM) have been shown to reduce plant dry weight by 18.15% to 39.67% and increase electrolyte leakage by up to 129.35%, underscoring the severity of metal-induced physiological disruption [26].

The downward movement of heavy metals through soil layers remains a serious concern because it can eventually contaminate groundwater, especially in areas affected by industry and mining [27]. In agricultural areas located near industrial zones, groundwater has been found to contain high metal levels; for example, Cr concentrations average around 19 µg L^−1^. Poor farming practices further intensify these issues. The Soil PLI shows much higher contamination in irrigated fields, where values reach 71.17, compared with 18.22 in rainfed systems, pointing to severe pollution under intensive irrigation [3]. These metals easily leach into groundwater largely depends on their chemical stability in the subsurface environment. In coastal aquifers, for example, the persistence of hexavalent chromium [Cr(VI)] can be influenced by the presence of borate ions [B(OH)_4_^−^]. These ions may act as buffers, helping to stabilise redox conditions and thereby affecting the long-term behaviour of Cr in groundwater systems [28].

Given that the accumulation of toxic metals in edible plant tissues represents a significant risk to the food chain, phytostabilisation strategies using metal-tolerant plant species and soil amendments are increasingly being explored [29]. Amendments such as humic acid and selenium, applied at 3 µM as a foliar treatment or 250 mg kg^−1^ in soil, have been shown to reduce root-to-fruit metal translocation and mitigate food safety risks [3,26]. To better illustrate how soil conditions and regional production systems influence Cd behaviour and its transfer to edible plant parts, Table 1 summarises reported patterns of Cd Phytoavailability, fractionation, and resulting concentrations in chilli fruits across different production regions.

### 2.2. Root Uptake and Translocation Pathways

The entry of Cd into the chilli plant’s system is a multi-stage process involving radical absorption, cellular compartmentalisation, and vascular transport. As a non-essential trace metal, Cd lacks dedicated transport proteins and instead hijacks the pathways utilised by essential micronutrients such as Fe, Mn, and Zn [21]. The accumulation patterns are highly cultivar-specific, with plants categorised into high-accumulation (e.g., X55), medium-accumulation (e.g., Daguo 99), and low-accumulation types (e.g., Luojiao 318) based on their physiological strategies for sequestration and fruit translocation [32]. The molecular regulation of Cd uptake is driven by a complex network of metal transporters. Studies in the Zunla 1 chilli pepper variety revealed that exposure to 50 µM Cd triggers massive transcriptomic shifts; 2451 differentially expressed genes (DEGs) were identified within 6 h (892 up-regulated, 1559 down-regulated), expanding to 3338 DEGs by 12 h (1766 up-regulated, 1572 down-regulated) [33]. Key transport families identified in these responses include the Natural Resistance-Associated Macrophage Proteins (NRAMP), Heavy Metal ATPases (HMA), and ATP-binding cassette (ABC) transporters [5,33]. In Capsicum tissues, HMA1, HMA2, and NRAMP1-6 are significantly up-regulated in roots and stems under stress [32]. Additionally, the phytochelatin synthase (PCS) gene expression follows a specific hierarchy among cultivars: X55 > Luojiao 318 > Daguo 99, suggesting that higher tolerance in varieties like X55 is linked to superior chelation capacity. Transcription factors from the ERF, WRKY, and NAC families, along with copper transport ATPases, serve as the primary regulatory nodes for these uptake pathways [5].

Water shortage causes profound changes in root structure and function, particularly by disrupting membrane integrity, which in turn affects how cadmium moves within the plant [34]. In Siete Caldos chilli pepper (*Capsicum frutescens*), a 14-day interruption of irrigation results in a clear decline in relative water content and membrane stability. These changes are accompanied by a rapid rise in electrolyte leakage, detectable as early as the third day of drought exposure [7]. At the molecular level, plants respond to this combined stress by activating defence pathways, including increased expression of antioxidant genes such as CuSOD, MnSOD, and CAT, along with P5CS, which supports proline synthesis and osmotic adjustment [7].

Drought conditions also influence the regulation of aquaporins, proteins that play a central role in water transport and may indirectly affect metal movement within plant tissues [35]. In the drought-tolerant genotype KCa-4884, all twelve analysed aquaporin genes were up-regulated under water stress, whereas the same genes showed reduced expression in the drought-sensitive G-4 variety [36]. These shifts in water permeability are often linked with enhanced antioxidant defences. For example, POD activity has been reported to increase between 1.9- and 3.6-fold as water stress intensifies [37]. In genotypes such as Ghotki, glutathione POD activity rose to 190 units during POD formation under drought conditions at 35% field capacity, reflecting a strategic shift in metabolism away from growth and toward stress protection [38].

Once Cd enters the root, its distribution follows a predictable subcellular hierarchy: cell wall (F1) > organelle (F2) > cell soluble fraction (F3). The cell wall acts as the primary barrier, isolating the majority of heavy metals to protect the protoplasts. However, the capacity of the cell wall to bind Cd is limited by the availability of functional bases in polysaccharides like pectin and hemicellulose [32].

Translocation from roots to aerial parts varies significantly by cultivar:

- X55 and Daguo 99: Primarily migrate Cd from roots to stems and leaves, effectively inhibiting migration to the fruit [32].
- Luojiao 318: Exhibits a higher migration coefficient to the fruit as soil Cd levels increase, despite having the lowest root Cd concentration [32].
- Zunla 1: Identified as a Cd hyperaccumulator where leaves serve as the primary accumulation sink [33].

Subcellular fractionation data reveal clear varietal differences in how Cd is partitioned within chilli fruits. When plants were exposed to 5 mg kg^−1^ Cd, the cultivar Daguo 99 accumulated substantially higher Cd levels in its fruit tissues than the other tested varieties. Specifically, Cd concentrations in the F1 fraction of Daguo 99 were 2.86 times higher than those in Luojiao 318 and 5.69 times higher than in X55. A similar pattern was observed at the higher exposure level of 10 mg kg^−1^ Cd, where Cd content in the F2 fraction of Daguo 99 fruits remained elevated, exceeding that of the other cultivars by a factor of 1.95 to 2.52. These results indicate that, although chilli plants generally possess some capacity to restrict Cd uptake, effective prevention of Cd accumulation in edible fruit tissues appears to be genotype-dependent. Cultivars such as X55 likely rely on more efficient vascular loading and transport control mechanisms, potentially regulated by the *FTP1-2* and *FTP1-3* genes, to limit Cd translocation to the fruit [32].

### 2.3. Fruit Bioaccumulation and Quality Trade-Offs

The final accumulation of Cd in the fruits of chilli pepper represents a critical juncture where plant defence mechanisms intersect with agricultural quality and food safety. While chilli is categorised as a Cd hyperaccumulator, with leaves serving as the primary sink, the partitioning of metals within the fruit and the resulting metabolic shifts dictate the economic and nutritional value of the crop [33]. In hot pepper genotypes such as *Pusajuala* and *Ghotki*, drought and metal stress typically decrease fruit pungency as energy is consumed by antioxidants like glutathione peroxidase (GPX), which can reach 190 units during stress [38]. Capsaicinoid accumulation normally reaches its maximum around 40 days after anthesis and gradually declines thereafter. However, the presence of heavy metals can hasten this decline by promoting POD-driven oxidation of phenolic precursors involved in capsaicinoid biosynthesis [39]. Elevated concentrations of Cd (≥5 mg kg^−1^) or Cu (≥100 mg kg^−1^) have also been shown to inhibit seed germination and reduce pigment levels in chilli plants. At the same time, these stresses stimulate the accumulation of soluble sugars and proline, which function as osmoprotectants and help maintain cellular stability. Excess Ni further disrupts nutrient balance by lowering the availability of essential elements such as N, K, and P within plant tissues [39]. The application of nano-selenium (nano-Se) at 5 mg L^−1^ has been shown to mitigate these trade-offs by inducing the phenylpropane-branched fatty acid pathway (*BCAT*, *Fat*, *AT3*, *HCT*, and *Kas*). This treatment significantly recovered and even enhanced metabolite levels, increasing capsaicin by 29.6%, nordihydrocapsaicin by 44.2%, and dihydrocapsaicin by 45.3% [40].

**Figure 1:**
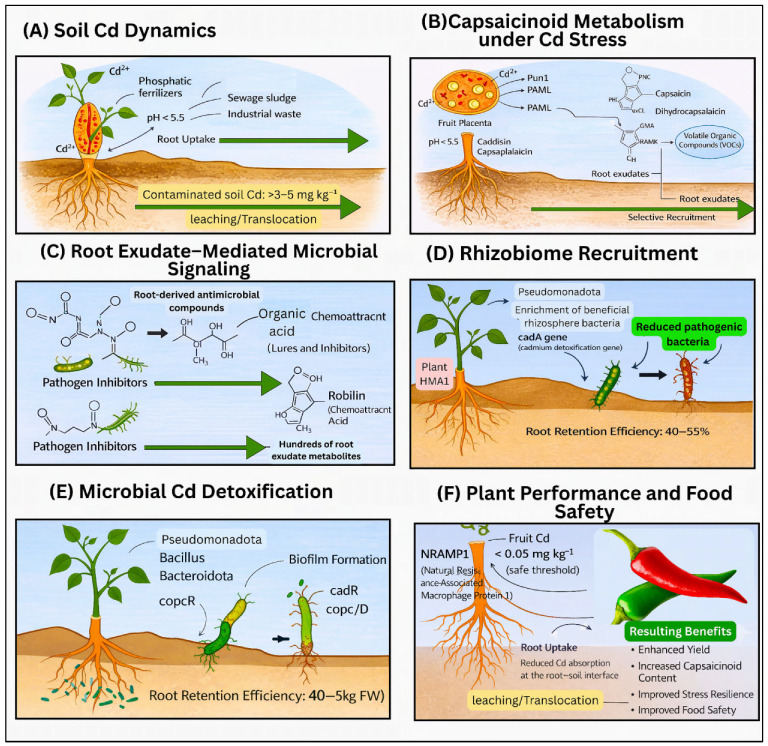
Conceptual overview of Cd dynamics, capsaicinoid metabolism, and rhizobiome-mediated stabilisation in chilli pepper. Panels (**A**,**B**) illustrate Cd sources, soil conditions, and capsaicinoid metabolic responses under Cd stress, while panels (**C**–**E**) summarise that root exudates and microbial functional traits are commonly associated with rhizosphere structuring, microbial detoxification processes, and root-level Cd retention. Panel (**F**) integrates these interactions to show coordinated plant–microbe responses are linked with reduced Cd translocation to fruits, improved plant performance, and enhanced food safety. Arrows indicate the direction of Cd movement, metabolic pathways, microbial interactions, and functional relationships among plant–soil–microbe components.

As shown in Figure 1, Cd behaviour in chilli agroecosystems is linked set of processes that begin with soil metal inputs and mobility and extend through root uptake, root exudate signalling, microbial detoxification, and rhizobiome recruitment. Together, these interactions determine the extent of Cd sequestration within plant tissues, influence capsaicinoid biosynthesis, and ultimately shape overall plant performance and fruit quality. The bioaccumulation of toxic elements in chilli increased the risk to human health, especially in regions with high soil metal accumulations [30]. In the Lanmuchang mining area, residents have been found to ingest 1.90 mg of Thallium (Tl) daily through their diet, resulting in urine Tl levels of 2.51–2.67 µg/L, well above the standard < 1.00 µg/L limit [25]. In dried chilli peppers from Guizhou Province, heavy metal concentrations follow the sequence: Cr > Pb > As > Cd [18]. Specific regional exceedances were noted in which Cr is reached 2.219 mg kg^−1^ in the AS region, exceeding permissible limits. And the Pb has surpassed the limits in the QDN region (1.894 mg kg^−1^), with 33% to 89% exceedance rates across several areas. Cd has exceeded limits by 214% in the QDN region (0.157 mg kg^−1^) and 130% in the LPS region [18].

## 3. Cultivar-Specific Capsaicin Variants

Capsaicinoid production is a defining feature of the *Capsicum* genus, responsible for the distinctive pungency that characterises chilli peppers. Beyond its importance for flavour and market value, this biochemical trait plays a key role in how the plant copes with environmental challenges such as heavy metal exposure and water scarcity [41]. Recent progress in high-resolution telomere-to-telomere (T2T) genome sequencing, together with multi-locus genome-wide association studies (GWAS), has shed new light on the genetic mechanisms underlying differences in capsaicinoid composition among chilli cultivars. These approaches are helping to clarify how genetic variation shapes both pungency and stress resilience in *Capsicum* [11,42].

### 3.1. Genetic Architecture of Pungency Traits

For many years, pungency in chilli pepper was thought to follow simple Mendelian inheritance, largely controlled by the Pun1 locus. More recent genetic studies, however, suggest that this view is overly simplistic. Research on varieties such as the Japanese cultivar ‘Shishito’, which is known for its extremely mild or nearly non-pungent fruits, shows that pungency is often a quantitative trait influenced by multiple genetic factors. In an F_2_ population generated from a cross between ‘Shishito’ and the highly pungent cultivar ‘Takanotsume’, capsaicinoid levels varied widely and were regulated by two major quantitative trait loci (QTLs). Together, these loci accounted for 39.8% and 19.7% of the observed genetic variation in pungency [17].

Phenotypic data confirms the strength of these genetic drivers. In homozygous ‘Takanotsume’ (TK/TK) alleles at *Shql3*, capsaicinoid levels were recorded at 17,817 to 18,245 µg·DW^−1^, whereas ‘Shishito’ (SH/SH) homozygotes at the same locus dropped to 7958–10,658 µg·DW^−1^ [17]. A genotypic shift from TK/TK to SH/SH resulted in a 41.6% to 55.3% reduction in capsaicinoids at the *Shql3* locus and a 36.7% to 59.7% reduction at the *Shql7* locus. Furthermore, GWAS on 123 pepper genotypes identified 30 single-nucleotide polymorphism (SNP) markers across 11 chromosomes influencing Scoville heat units (SHU), with markers on chromosome 3 explaining up to 58.71% of the variation for capsaicin content [33].

The Pun1 locus (also referred to as *AT3* or *CS*) remains the most significant qualitative regulator of pungency. It encodes capsaicin synthase, the enzyme responsible for the final condensation step of the capsaicinoid pathway. Allelic diversity at this locus often dictates the presence or absence of heat [42].

- pun1: A 2.5 kb deletion spanning the promoter and first exon found in non-pungent cultivars like ‘Jupiter’ and ‘Maor’, resulting in a total lack of transcription.
- pun1^2^: A four-base pair deletion causing a frameshift and a truncated, non-functional protein, characteristic of the non-pungent NMCA30036.
- pun1^3^: Observed in *C. frutescens* PI594141, containing insertions/deletions that truncate the second exon [10].

T2T gapless genome assemblies of pungent *C. annuum* (CaT2T) and wild non-pungent *C. rhomboideum* (CrT2T) provide a high-resolution view of these regions. CaT2T encodes 117 putative capsaicinoid biosynthesis genes (CBGs), including tandem duplications of *AT3* (*AT3-1* and *AT3-2*). While *AT3-1* is functional and associated with pungency, *AT3-2* is a pseudogene. In non-pungent *C. rhomboideum*, the loss of pungency is associated with substantial genomic divergence, including 10 fissions and 11 fusions of chromosomes compared to the *C. annuum* karyotype [11].

The genetic regulation of capsaicinoids is intrinsically linked to hormonal signalling and environmental context. Soil quality plays a vital role in this regulation; for instance, capsaicin content and capsaicin synthase activity were significantly higher in endemic chillies grown in alluvial soil compared to lateritic soil [43]. Interestingly, while the Pun1 gene was upregulated in lateritic soil, pungency was ultimately inhibited by the overexpression of the recessive Pun12 allele [13]. This suggests that high organic Carbon, microbial activity, and NPK status in alluvial soils act as suppressors for recessive non-pungent alleles. Furthermore, the “pungency-variable” nature of the ‘Shishito’ pepper provides evidence of a link between seed development and metabolite accumulation. Few-seeded fruits exhibit higher fluctuations in capsaicinoid content than many-seeded ones [44]. Expression analysis of 18 genes revealed that 11 of them, including *CaMYB31*, *WRKY9*, *BCAT*, *FAT*, and *pAMT*, show significant positive correlations (*p* < 0.01) with capsaicin concentration. In high-pungent fruits, these genes are significantly upregulated, whereas they remain at low expression levels (less than 500 µg·gDW^−1^) in low-pungent variants [45]. Hormonal crosstalk, particularly through the ethylene-responsive transcription factor (ERTF) and putative acyl-activating enzyme 2, has been identified via GWAS as a candidate mechanism for regulating Scoville heat units [42]. Additionally, mRNA-miRNA integration studies show that the plant-hormone signal transduction pathway, involving 40 differentially expressed genes (e.g., *SAUR32L*, *EIN2*, *IAA*), is central to the heterosis and development of *Capsicum* fruits [44].

The spatial and temporal specificity of capsaicinoid biosynthesis is governed by placenta-specific open chromatin regions (OCRs). Multi-omic profiling using CaT2T as a reference identified that *CS-2*, *MYB31*, and *MYB48* are specifically expressed in the placenta, facilitated by OCRs with low methylation levels within 2 kb upstream of their start sites. Sequence motif enrichment analysis revealed five transcription factor binding motifs (TFBS), MYB, G-box, Box-4, ABRE, and MYC, that are conserved across major CBGs like *CS*, *ACL*, *KasI*, and *BCAT* [11]. Beyond the Pun1 and *Pun3* loci, recent research has characterised the *Pun4* locus on chromosome 6. Induced mutants (‘221-2-1a’ and ‘1559-1-2h’) derived from the pungent ‘Yuwolcho’ landrace showed a drastic reduction in capsaicinoids, dropping from 20,979.2 µg/g DW in the wild type to as low as 17.7–258.8 µg/g DW in the mutants. The *Pun4* locus was mapped to a 208-Mb region, with *DEMF06G16460* (encoding 3-ketoacyl-CoA synthase) identified as the primary candidate gene. In these mutants, the expression of genes associated with the branched-chain fatty acid pathway, such as *BCKDH*, *ACL*, *KasI*, and *FAT*, was significantly suppressed [12].

The evolution of these pathways is further clarified by the study of *pAMT* (vanillylamine synthase). Phylogenetically, *pAMT* is a member of the Solanaceae cytoplasmic GABA-transaminases (GABA-Ts), likely originating from a duplication of a chloroplast ancestor. While plant GABA-Ts generally possess the ability to produce vanillylamine, *pAMT* in *Capsicum* evolved a higher catalytic efficiency (lowest Km value) and exclusive expression in the placental septum, establishing the unique pungency of the genus [46]. Capsaicinoid biosynthesis in chilli peppers converts the branched-chain fatty acid pathway (to branched acyl-CoA moieties) with the phenylpropanoid pathway (to vanillylamine). Capsaicin, synthesised by the Pun1 locus capsaicin-synthase in the placenta, is the condensation of these precursors. Loss-of-function mutations in Pun1 and Pun4 have been shown to cause changes in the major steps of the process, causing cultivar-dependent losses in pungency.

### 3.2. Comparative Profiling: ‘Bhut Jolokia’ vs. ‘Guntur Sannam’

The genetic architecture and the agro-climatic environment of the geographical origin of Capsicum cultivars are the main factors that contribute to the metabolic and sensory divergence between different cultivars [47]. Within the high-pungency cultivar group, two extremes of capsaicinoid concentration and chemical complexity are represented by Bhut Jolokia (of the C. Chinese lineage) and Guntur Sannam (a popular Indian *C. annuum* type, commonly known as the Indian pepper type or Guntur type). It is necessary to understand their relative profiles, including SHU to the complex rhizospheric metabolome, to explain how these plants regulate their environment to stabilise heavy metals and protect against pathogens defense [31,48]. The economic and functional importance of these peppers is essentially linked to their SHU that is obtained by the exact concentrations of capsaicin and dihydrocapsaicin [49]. The comparative studies show that Indian pepper (Guntur type) is characterised by high pungency, and its SHU are 85,909 in powdered form, and the overall capsaicinoid contents are 5.571 ± 0.139 g kg^−1^ [48]. On the contrary, some cultivars like ‘Khyati’ (KT), a popular Indian type, have a pungency of 16,526 SHU, which is backed by the levels of capsaicin and dihydrocapsaicin of 499.19 mg kg^−1^ and 418.93 mg kg^−1^, respectively [31]. High-heat varieties have a pungency that is defined by a surface form of stimulation, which sensory panellists have described as scorching, heatwave-like, and searing sensations that run throughout the oral cavity. These extreme peppers have an explosive sensory reaction, where the latency duration is 0–3 s, and the duration is 3–5 min [48]. Capsaicinoids have significantly higher extraction efficiency and thermal stability in oil matrices than in powder matrices. Dihydrocapsaicin has a lower rate of degradation in oil than capsaicin because of its increased molecular saturation and thermostability. Even though capsaicin and dihydrocapsaicin jointly contribute to about 90% of the perceived pungency, there is a strong correlation between their concentrations (r = 0.914 in powders and r = 0.947 in oils), which proves the reliability of the two compounds as the main predictors of scaling the intensity of heat [48].

The rhizosphere volatilome, which is the total profile of volatile organic compounds (VOCs), is a unique biochemical fingerprint of chilli growing varieties and is highly influenced by soil composition and agro-climatic conditions [50]. Hydrocarbons (n = 36) are the most common VOC class, followed by aldehydes (n = 12) and esters (n = 10). Linalool is a terpene alcohol produced through the methylerythritol phosphate pathway and is a ubiquitous VOC that provides typical aroma profiles and antimicrobial properties [31]. Certain VOCs are biomarkers of high-pungency cultivars. While VOCs help distinguish chilli varieties based on aroma and ecological interactions, direct measurements of capsaicinoid concentration and heat intensity provide a quantitative basis for comparing pungency across cultivars. In the KT type, a-phellandrene and bicyclohexane, 4-methylene-1-(1-methylethyl)-, produced by the terpenoid biosynthesis (TpB) pathway, have been associated with high heat intensity. Conversely, the Jwala type is typified by the substitution of phenylalanine with phenylalanine 2-methoxy, the product of the shikimate pathway that confers a pleasant aroma and plays a role in the hydrophobic gloss formation of the fruit cuticular wax [31]. The use of insect-derived residual streams has been reported to improve soil fertility parameters, such as total organic carbon, total nitrogen, total phosphorus, and total potassium, and significantly increase enzyme activities, such as catalase, b-glucosidase, and urease [51]. These enzyme improvements show positive associations (r > 0.49) with the growth characteristics of plants, such as plant height (up to 45.0 cm in treatment groups) and fresh biomass gains up to 145% [52].

### 3.3. Rhizobiome Response to Pungency Gradients

Bacterial communities associated with pepper roots are largely dominated by members of the phyla *Pseudomonadota* and *Bacteroidota*, which together make up about 91% of the total microbial population. Fine-scale spatial analyses conducted at the millimetre level show that these communities are not uniformly distributed along the root surface. Instead, their composition changes gradually with distance, displaying a moderate distance decay pattern, with community dissimilarity ranging from as little as 2.4% to as much as 96.9% along individual roots [15]. Microbial richness and diversity are significantly influenced by the plant’s “economic strategy”—a gradient from fast (FES) to slow (SES) growth. Varieties classified under the Slow Economic Strategy (SES) exhibit a significant decrease in microbial richness compared to FES and Medium Economic Strategy (MES) plants [25]. Notably, SES plants show a stronger correlation between rhizosphere microbial diversity and functional traits, such as root biomass and nutrient accumulation, compared to FES varieties. In comparisons between healthy and diseased plants, healthy rhizospheres exhibit significantly higher bacterial richness (*p* < 0.05), with Alphaproteobacteria and Actinobacteria being consistently associated with disease-suppressive soil environments [53].

**Figure 2:**
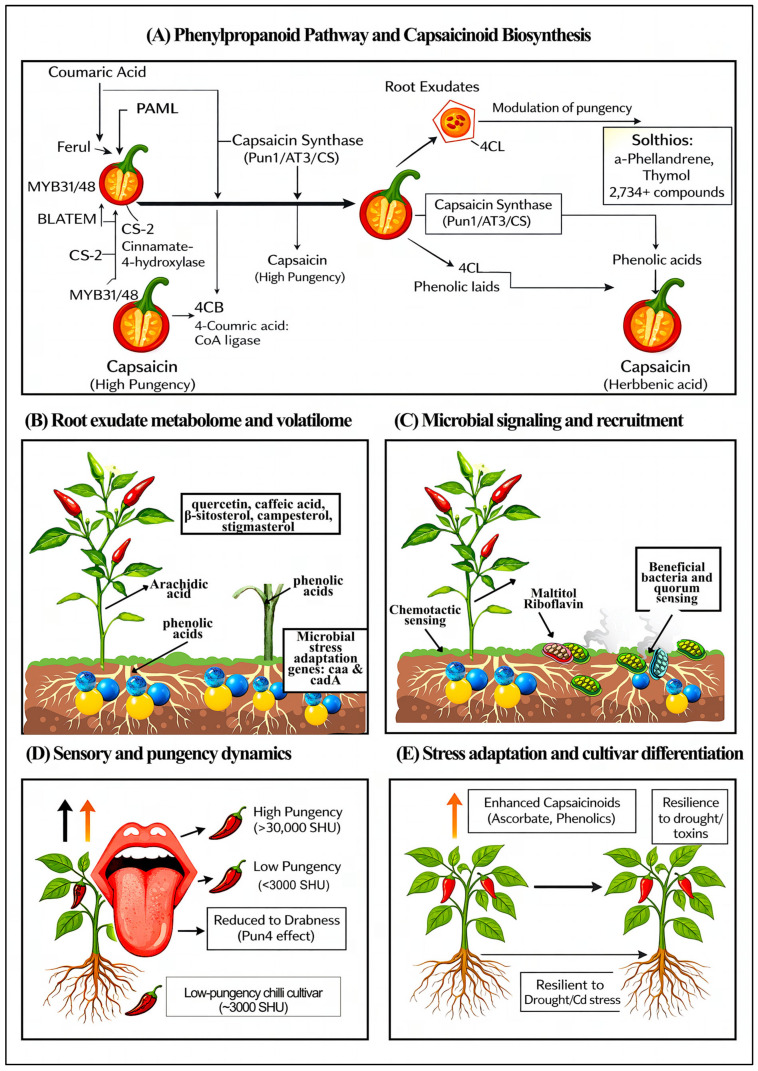
Integrated overview of capsaicinoid metabolism, root exudation, and rhizosphere interactions in chilli pepper. Panel (**A**) outlines the phenylpropanoid and capsaicinoid biosynthetic pathways and key regulatory genes involved in pungency formation. Panel (**B**) depicts the complex composition of root exudates and volatile organic compounds released into the rhizosphere, including phenolic acids, sterols, and other secondary metabolites. Panel (**C**) illustrates microbial sensing and enrichment patterns commonly observed in association with these chemical gradients, highlighting the prevalence of plant growth–promoting and stress-tolerant taxa. Panels (**D**,**E**) summarise how differences in capsaicinoid levels among cultivars are associated with variation in pungency perception, microbial assemblages, and physiological responses related to cadmium retention and drought tolerance. Overall, the figure integrates evidence from multiple studies to show how cultivar-specific metabolic traits coincide with rhizosphere structuring and stress adaptation outcomes.

As illustrated in Figure 2, variation in chilli pungency is closely associated with coordinated changes in phenylpropanoid and capsaicinoid biosynthesis, root exudate composition, and microbial signaling in the rhizosphere. High-pungency plants channel carbon toward capsaicin synthesis through tightly regulated genetic networks (e.g., Pun1, *AT3*, *CS*), while simultaneously releasing phenolic acids, flavonoids, and sterols into the soil that are consistent with a role as chemical cues associated with microbial chemotaxis and enrichment. These exudates are associated with shifts in rhizosphere community structure, often correlating with higher relative abundances of beneficial bacterial groups involved in stress tolerance, detoxification, and quorum sensing, whereas shifts toward lower pungency are associated with altered exudate profiles, reduced microbial specialisation, and greater susceptibility to biotic and abiotic stress.

Chilli peppers host a diverse rhizobiome that lives within internal tissues without harming the host, acting as a “hotspot” for synergistic interactions [52]. Colonisation efficiency is often improved through intercropping or the application of non-native consortia. Intercropping chilli with Chinese chives (*Allium tuberosum*) is associated with reorganises the endosphere. In this system, 69.54% of the microbial communities in Chinese chive roots originate from pepper roots, illustrating high cross-host transfer [54]. Non-native endophytes like *Streptomyces panaciradicis* (MR13) and *Bacillus subtilis* (MR3) demonstrate high colonisation potential. Mixed cultures of these strains have increased seedling height to 17.0 cm compared to 12.6 cm in controls [55]. High-efficiency endophytes such as MR10 and MR13 have achieved vigour indices (VI) as high as 12,457, and seed germination energy reaching 221% [55]. The endospheric community of diseased plants is characterised by lower complexity and stability compared to healthy plants. In diseased tissues, the genus *Fusarium*, a primary agent of pepper wilt, can increase in abundance by 2-fold, while plant fresh weight decreases by 72% [53].

The enrichment of specific taxa is accompanied by an enrichment of functional genes related to plant growth promotion and stress resilience. Functional genes for P solubilization (55–88 mg L^−1^), indole-acetic acid production (20–164 mg L^−1^), and ACC deaminase production (0.317–0.375 mM) are prevalent among recruited endophytes [55]. Beyond microbial functional capacity, cultivar-specific metabolic traits are associated with differences in the enrichment and activity of beneficial taxa. In particular, variation in capsaicinoid production among chilli genotypes reflects differences in root-derived chemical signaling that is correlated with microbial enrichment patterns. Accordingly, Table 2 presents reported capsaicinoid levels for representative cultivars, providing metabolic context for the functional gene enrichment observed in recruited rhizosphere and endophytic communities. In healthy endophytes, there is a distinct enrichment of pathways related to the biosynthesis of other secondary metabolites, which is absent in diseased plants. However, intercropping optimises functional synergy; the proportion of positive microbial interactions in pepper leaves increases to 90.45%, while negative interactions of *Bradyrhizobium* decrease by 97% [54]. Recruited taxa like *Sphingobium*, *Steroidobacter*, and *Humicola* enhance carbon and nitrogen cycling functions (e.g., chitinolysis and cellulolysis), that are associated with reduced prevalence of persistent pathogens such as *Phytophthora capsica* and *Colletotrichum* species [51].

## 4. Rhizobiome Engineering Mechanisms

The chilli pepper rhizosphere is a very dynamic ecological stage where the secondary metabolic product of the plant, namely the pungency gradient formed by capsaicinoids, interacts with multifaceted microbial communities. These interactions cause niche differentiation and determine the recruitment of beneficial and pathogenic taxa, which ultimately determine the pathological condition and economic strategy of the plant [59,60].

### 4.1. Capsaicin as a Selective Microbial Signal

Capsaicin is a very sensitive biosynthetic compound to the soil environment. The capsaicin synthase activity and capsaicin content of alluvial soils with high content of organic carbon and microbial activity are much greater than those of lateritic soils. This change in metabolism is associated with by the increased expression of genes like Csy1, which has a higher relative quotient value in alluvial soil (Nd = 0.961) than in lateritic soil (Nd = 0.571). The resulting capsaicin gradient is consistent with a filtering role associated with the enrichment of specialised rhizosphere microbial communities [13]. Chilli varieties of high pungency, which belong to particular growth strategy groups including the SES, exhibit a stronger and more stable relationship between their functional properties and rhizosphere microbial communities [61]. Selective signalling of capsaicin and its metabolic precursors promotes the recruitment of Cd-resistant and plant growth-promoting taxa. The strains, such as *Priestia megaterium* KW16, which are found in polluted environments, are extraordinarily resistant to high levels of Zn, Cu, Ni, and Cd [60]. These strains are frequently associated with chemotactic responses to specific root exudates, facilitating their enrichment in the root zone. In healthy chilli plants, the genus *Streptomyces* and *Bacillus* are enriched, which promotes the production of secondary metabolites that increase the resistance of the host [51]. In particular, the central ASVs in SES varieties are strongly positively correlated with nutrient uptake, such as P uptake, which is necessary to relieve metal toxicity [61].

The *Capsicum* volatilome, comprising over 2734 identified VOCs, serves as a complex signalling system associated with long-distance microbial and ecological interactions [14]. These volatile signals are essential for inducing systemic resistance (ISR) and regulating defence-related genes. VOCs produced by rhizobacteria such as *Pseudomonas fluorescens* PDS1 and *Bacillus subtilis* KA9 significantly increase the activity of antioxidant enzymes like superoxide SOD and POD in chilli plants. In leaf tissues, the maximum POD activity reached 0.090 ± 0.002 ΔOD min^−1^ mg^−1^ when treated with a combination of these volatile-producing strains [62]. These VOCs linked to enhanced expression of key antioxidant and defense genes, including PAL, POD, SOD, WRKY40, and NPR1. Interestingly, the overall gene expression is typically higher in root tissues than in leaf tissues, emphasising the root-centric nature of volatile signalling. Volatile-mediated interactions do not require physical contact, allowing strains like *P. megaterium* KW16 to inhibit the growth of pathogens like *Rhizoctonia solani* by up to 58% through the air [61]. Common volatile markers in *Capsicum* systems include azulene, dodecane, and various benzene derivatives, which serve as indicators of the plant’s health and stress status [62].

The effective colonisation of endophytes in the Capsicum tissues is a dose- and method-specific phenomenon that determines the effectiveness of the promotion of plant growth and suppression of plant diseases. Foliar spraying was observed to be the most effective inoculation method in *Beauveria bassiana* studies, as compared to soil drenching and seed immersion [63,64]. Entomopathogenic fungi re-isolated on pepper leaf develop mycelium fully in 11 days at 25 °C and 95% humidity [65]. In terms of pest control, *B. bassiana* caused 100% aphid mortality rate in 21 days at high levels of conidial suspensions (10^8^ conidia mL^−1^), which reduced the pest population by 68 individuals in control plants (0.7 ± 1.2) individuals in colonised plants [65]. Endophytic colonisation is also strongly correlated with increased plant biomass. The growth of inoculated oilseed rape plants with *Paenibacillus megaterium* KW16 showed dramatic growth improvements, with shoot biomass increasing by 216% and root biomass by as much as 1737% compared with pathogen-infected control plants [60]. In chilli, useful endophytes obtained in various sources, such as neem and moringa, can enhance relative chlorophyll content (maximum of 40.3 SPAD value) and seedling height (maximum of 17.0 cm vs. 12.6 cm control). These responses are further optimised by the synergistic interaction between endophytes and arbuscular mycorrhizal fungi (AMF). The maximum AMF colonisation rates are seen in chilli peppers when irrigation is reduced, and AMF inoculation increases the flavonoid content and decreases the oxidative stress markers, increasing plant tolerance to abiotic stress [66,67]. Capsaicinoid root exudates generate a pungency gradient that is associated with selective enrichment patterns of metal-resistant and plant growth-promoting bacteria, contributing to niche differentiation and reduced dominance of pathogenic taxa. Enriched genera: High-pungency varieties have a positive relationship with augmented phosphorus uptake and mitigation of metal toxicity in the host plant: Streptomyces and Bacillus are enriched [13,61]. It should be noted that much of the current evidence linking capsaicinoid levels with microbial recruitment is based on correlative field and greenhouse studies. While chemotactic responses to root exudates and consistent enrichment of specific taxa (e.g., Streptomyces and Bacillus) support a signalling role for capsaicinoids, direct mechanistic validation using capsaicinoid-deficient mutants, targeted exudate manipulation, or isotope-tracing approaches remains limited.

**Figure 3:**
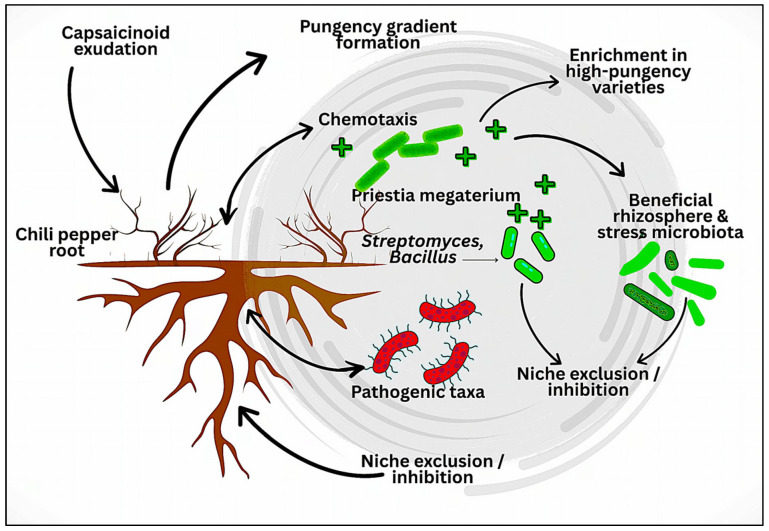
Conceptual model of capsaicinoid-associated rhizosphere structuring in chilli pepper. The figure illustrates how capsaicinoid exudation from chilli pepper roots may contribute to the formation of a pungency gradient in the rhizosphere, associated with the enrichment of beneficial and stress-tolerant microbial taxa (e.g., *Priestia*, *Streptomyces*, and *Bacillus*) and the suppression of pathogenic taxa through niche differentiation and competitive interactions.

This process is the basis of designed rhizobiome assemblage in *Capsicum* species (Figure 3).

### 4.2. Cd-Sequestration Endophyte Functions

The development of climate-resistant rhizobiomes is largely dependent on endophytic bacteria that can capture and neutralise toxic metals such as Cd. In addition to active efflux, endophytes also use a complex set of genomic and biochemical processes to prevent translocation of toxic metal ions within the root system to the edible aboveground tissues by passive sequestration on the cell wall and extracellular matrix [57,58,68]. The genomic landscape of metal-resistant endophytes reveals a robust selection of efflux systems designed to maintain cellular homeostasis under Cd stress. Sequencing of endophytic *Pseudomonas* sp. strains (AM4, AM8, AM14, Z13, Z18) showed draft genome lengths ranging from 6.1 Mb to 7.4 Mb, with a mean G + C content of 60–61% and between 5700 and 7059 predicted coding sequences [58]. These genomes encode the Czc system, a complex cation–proton antiporter that actively transfers Cd^2+^, Co^2+^, and Zn^2+^ ions out of the bacterial cell. Key components identified include:

- czcD: Encodes a cation diffusion facilitator transporter.
- czcB: A critical component of the CzcCBA efflux transporter system.
- czcR/czcS: A two-component regulatory system that modulates the expression of the Czc operon.

In addition to the Czc system, these endophytes carry cadA, which encodes a metal-translocating P-type ATPase that catalyses the active efflux of Zn, Cd, and Pb ions. Transcriptional regulators such as cadC and cadR further refine the bacterial response to Cd exposure [58,69]. The energy-dependent efflux of toxic ions represents the largest group of resistance systems in bacteria, effectively cracking the “toxic trap” of contaminants [70,71].

The single isolate DG-20 (*Curtobacterium oceanosedimentum*) was highly tolerant to Cd concentrations up to 18 mM and exhibited bioaccumulative removal capacity [57]. In chilli pepper, Cd is mainly concentrated in the root cell wall, and then in the cytoplasm and organelles. Subcellular examination of the Layan 101 type suggests that the main location of sequestration is the cell wall [72]. This binding is regulated by endophytic activity and exogenous signals such as abscisic acid or Boron. The application of Boron leads to a significant increase in the contents of lignin and pectin in the root cell walls that is a good inhibitor of Cd influx. The root tip meristematic zone has the highest Cd^2+^ flux, which is inhibited by the supply of Born [73,74]. Cd tends to accumulate in specific components of the root cell wall, particularly in the hemicellulose fraction. In chilli varieties such as ‘Layan 101’, Cd levels in hemicellulose 1 range from 2.91 to 17.72 mg kg^−1^, while slightly higher concentrations, from 5.81 to 20.83 mg kg^−1^, have been reported in ‘Layan 201’ [74]. Silicon plays an important role in strengthening this retention mechanism. When silicon is available, a larger share of Cd is locked into the root cell wall, an increase of about 23.2% to 24.0%, which limits its movement into the cell interior and helps protect sensitive cellular processes [75].

The association of the Cd-tolerant endophytes with the host plant antioxidant system leads to a synergistic defence system. The *C. oceanosedimentum* DG-20 inoculation of pot experiments increased chilli root and shoot lengths by 58% and 60%, respectively, without stress. The root length improved by 86% under Cd stress. Photosynthetic pigments (chlorophyll a and b) increased by up to 55.54%, and ascorbic acid levels significantly rose to 248.06 ± 3.62 ug/g FW in T1 treatments inoculated plants [57]. Proline content, which is an indicator of stress tolerance, rose to 4.22 ± 0.13 µg g^−1^ FW in plants that were treated with bacteria [57]. Cd was highly phytostabilized by both control and inoculated plants in roots (0.93 ± 0.12 µg mL^−1^) than in shoots (0.57 ± 0.07 µg mL^−1^), which is why the combination of chilli cultivars and specialised endophytes is an effective phytostabilisation method to reduce the probability of fruit contamination [57,75]. In the long-term, these endophytes and systemic signals induce the production of phytochelatins (PC2 and PC3) in the cytosol. In addition, e.g., enhanced PC2 and PC3 by 10-fold and 29-fold, respectively, relative to Cd-alone treatments, which facilitated vacuole compartmentalisation as a secondary defence line [75].

### 4.3. Plant and Microbe Mechanisms for Cadmium Phytostabilisation

It has been shown that the combination of functional bacterial agents, structured organic composites, and custom doses of nutrients could greatly increase the phytostabilisation ability of chilli peppers in polluted agroecosystems [76,77]. Accuracy in Cd dosing and experiment design is essential in deducing functional regression models to estimate food chain contamination. The background levels of Cd in typical Chinese soil systems are between 0.14 and 0.45 mg kg^−1^ (mean 0.27 mg kg^−1^) of total soil Cd [6]. The typical pot experiments that are planned based on Soil Environmental Quality Standards are control (CK), low Cd, and high Cd treatments. Bioavailability measurements indicate that EDTA-extractable Cd is 18.23–71.12% (mean 45.25) of total soil Cd, and strongly acidic soils (pH 5.3) have a high correlation (R2 = 0.8162) with total Cd content. Soil pH is a primary determinant of Phytoavailability, where strongly acidic soils (pH 5.3) have a high correlation with total Cd [6,78].

The root system has a physiological barrier effect, which is the main defence mechanism against metal translocation to the fruit. The accumulation experiments show that the levels of Cd in chilli pepper are strictly hierarchical: root > stem > fruit [6]. The bioaccumulation factor (BCF) of roots is 0.597–2.946, and the bioaccumulation factor of the fruits is much lower, with a range of 0.033–0.184. It is a high root retention efficiency, which tends to trap 40–55% of the loaded metal [6]. The root Cd levels are observed to rise between the average level of 0.41 mg kg^−1^ in CK treatments and 3.10 mg kg^−1^ in high-level Cd stress treatments. This is further boosted by inoculation with Cd-tolerant *Curtobacterium oceanosedimentum*, which prompts a 58 and 60% increase in root and shoot lengths, respectively, and an increase in the activity of the antioxidant system [57]. DTPA-extractable Cd in soil can be reduced by 53.1% with the use of coupled BBC-BS agents with smooth vetch residues, which is 37.3 times more effective than the individual treatment [78].

Phytostabilisation over a long period requires the stability of the recruited rhizobiome in the presence of toxic stress. The best efficacy has been demonstrated by heavy-metal-tolerant plant PGPR consortia, including *Pseudomonas azotoformans* (Pa), *Serratia rubidaea* (Sr), *Paenibacillus pabuli* (Pp), and *Bacillus velezensis* (Bv) in reducing metal translocation [79]. Consortia treatments (Pa + Sr + Pp + Bv) reduced Cd and Ni contents in leaves by 88.0–88.5% and 90.2–90.9%, respectively. In fruits, the Cd and Ni reductions were 87.2–88.1 and 92.3%, respectively. These microbial interventions enhanced chlorophyll a content and stomatal conductance by 125–129% and 88–96%, respectively, relative to non-inoculated controls subjected to heavy metal stress [79]. The exogenous biostimulants of garlic + onion extract with bee honey reduced the electrolyte leakage by 52% and malondialdehyde content by 49% and reduced the levels of superoxide and H_2_O_2_ by approximately 60% [80].

**Figure 4:**
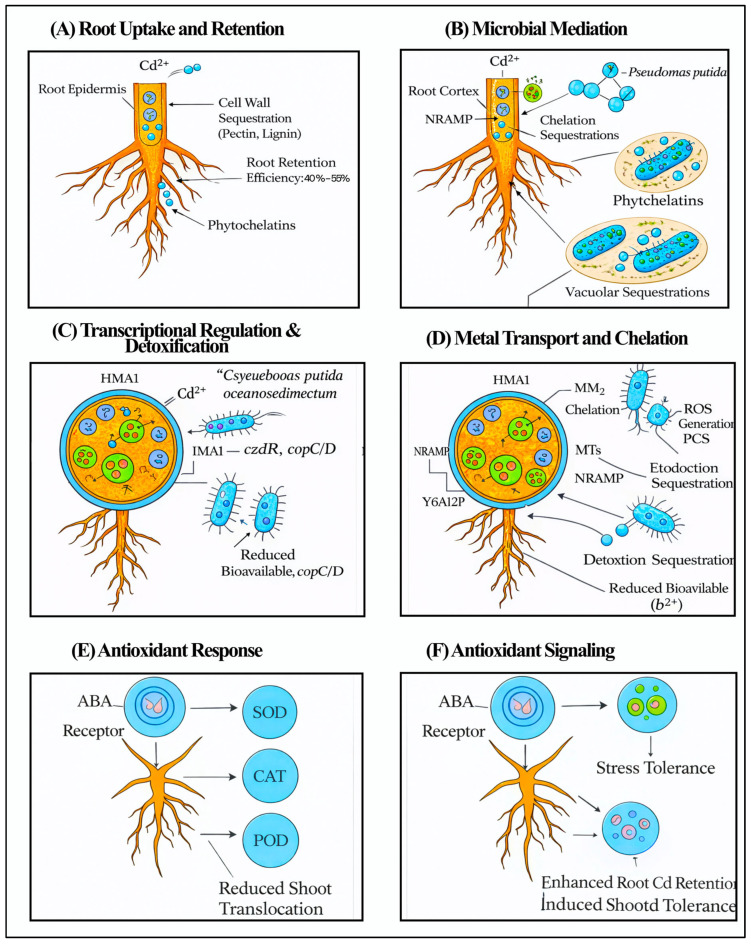
Conceptual overview of Cd uptake, retention, and detoxification processes in chilli pepper roots and associated microbial interactions. Panels illustrate (**A**) Cd uptake and retention in root tissues via cell wall binding and phytochelatin sequestration, (**B**) microbial mediation of Cd chelation and vacuolar compartmentalisation, (**C**,**D**) transcriptional regulation and metal transport mechanisms that reduce Cd bioavailability, and (**E**,**F**) antioxidant and stress-signalling responses contributing to improved Cd tolerance. Together, the figure summarises experimentally reported and correlative pathways by which plant physiological responses and rhizosphere microbes are associated with reduced Cd translocation and enhanced stress resilience.

*Bacillus* sp. LBF-01 showed a 9.04-fold reduction in disease occurrence caused by *Fusarium oxysporum*, improving seedling vigour by 592.8%, and facilitating sustainable production in contaminated soils [81]. The combination of these measures implies that the engineering of a robust rhizobiome by means of specific microbial recruitment and controlled organic amendments (e.g., 2% poultry manure biochar, which increases the number of fruits by 84.6) is the key to protecting the productivity of chilli pepper under the dual pressure of Cd toxicity and tropospheric ozone damage [82]. Figure 4 illustrates how cadmium is immobilised at the root level through cell wall binding, phytochelatin chelation, and vacuolar sequestration, thereby limiting its movement to shoots. Recruited PGPR further enhance this protection by lowering Cd bioavailability, regulating metal transport, activating antioxidant defences, and improving plant physiological recovery under metal stress.

## 5. Environmental Resilience and Trade-Offs

The success of *Capsicum* plants in farming depends on how well they can handle more than one stress at the same time. Studies on semi-domesticated and improved chilli pepper varieties show that the way these plants produce capsaicin is closely linked to how they respond when water is limited. The system that controls pungency also helps the plant deal with drought conditions [7,83].

### 5.1. Drought Synergies with Capsaicin Signalling

Drought stress serves as a strong trigger for changes in chilli peppers, often enhancing the production of secondary metabolites that enhance the defence system. Comparative evaluations of *C. annuum* and *C. chinense* demonstrate that while drought negatively impacts primary morphological traits, it triggers a significant proportional increase in phytochemical components such as total phenolics (TPh) and total flavonoids (TFv) [83]. In *C. annuum*, TPh reached maximum values of 6.02 mg GAE g^−1^ FW under severe stress (25% field capacity), while total antioxidant activity peaked at 26.85 µmol TEAC g^−1^ FW [40].

The chilli peppers use quick physiological adaptations to ensure water moves inside their tissues in the drying of the soil. In *C. frutescens*, stopping irrigation for 14 days caused strong physiological stress, but the plants did not reach permanent wilting. Because of this, all plants were able to survive once water was supplied again. The measurement data show that RWC starts decreasing on day 3 (about 8 ± 2%), and on day 14, it has reached critical levels of 30% in both leaf (51.36%) and root (54.96%) tissues [7]. This loss of water pressure was linked with a sharp increase in electrolyte leakage, which increases to 58.01% in drought-stressed aerial tissues as compared to 19% in control plants. Stomatal conductance was the most affected process, although treatment with salicylic acid at 1 mM helped the plants recover by supporting higher electron transport rates [84].

The buildup of osmoprotectants, especially proline, plays an important role in capsaicinoid biosynthesis. The high increase in Proline in *C. frutescens* in drought stress increases to 1489.27 mg g^−1^ fresh weight in leaves and 1219.76 mg g^−1^ fresh weight in roots. And these values are about 21 times higher in leaves and 17 times higher in roots compared to well-watered plants [7]. Pungency changes are a reflection of this osmoprotective response. Drought conditions in the hot chilli cultivar Jolokia cause a substantial rise in capsaicinoid levels and SHU, especially during short-term water stress lasting about 21 days [16]. This response is also supported by genetic evidence linked to sound and mechanical signals in plants. Under moderate water stress, specific vibration patterns were found to increase capsaicin levels by nearly six times by influencing the at3 and kas genes [37]. In addition, drought tolerance improves when the chloroplast-targeted chaperone protein AdDjSKI is more active. This protein helps protect photosystem II, allowing photosynthesis to continue and reducing cell damage, as shown by lower malondialdehyde levels [1].

The change from drought to rewatering highlights how flexible *Capsicum* plants are. Although chlorophyll levels drop sharply during drought, by about 90% after 14 days, these plants recover very quickly once water is restored. Within just 24 h of rehydration, chlorophyll levels double, rising from about 14.01 mg mL^−1^ to 28.31 mg mL^−1^ [7]. The antioxidant defence system also maximises the water-use efficiency. The highest activity of the antioxidant enzymes CAT and POD was observed on the 14th day of drought stress. POD activity reached 63.22 U mg^−1^ protein in the roots, while in control plants it was only 6.41 U mg^−1^ protein [7]. Such enzymatic benefits are essential in the preservation of cell structures in intermittent drought. Signalling molecules such as H_2_S and H_2_O_2_ help keep reactive oxygen species under control. They balance harmful molecules like superoxide and hydrogen peroxide, while also supporting normal glucose metabolism [85].

Beyond drought-related recovery and antioxidant responses, *Capsicum* plants exhibit broad physiological plasticity under multiple environmental stresses, including heavy metal toxicity and water limitation. Table 3 compiles key physiological and biochemical parameters affected by Cd, drought, and hydric stress, and illustrates how microbial consortia, rehydration strategies, and other mitigation approaches improve plant performance and stress tolerance.

### 5.2. Quality and Safety of Fruits

The ultimate metabolic and safety profile of Capsicum fruits in a stressed environment is the ultimate measure of phytostabilisation effectiveness. The efficiency of heavy metal exclusion and preservation of high-value bioactive compounds is determined by genetic diversity in indigenous and commercial cultivars. Recent pangenomic and phenotypic analyses of various germplasm—including the five domesticated species—have shown complicated polygenic designs that regulate these quality attributes under stress [86]. The Cd distribution inside the fruit is important for food safety because the pericarp is the main edible part [87]. Genome-wide association studies using the *C. annuum* reference genome and a multi-species pangenome have identified many key genetic markers linked to fruit quality and how minerals are stored. In total, 144 and 150 important SNPs were associated with these traits [86]. Several genes involved in cell wall structure, such as galacturonosyltransferase 12 on chromosome 3 and fasciclin-like arabinogalactan proteins on chromosome 9, help lock metals into the fruit’s structural tissues. This reduces the amount of soluble Cd in the edible pericarp. The Cd accumulation in fruit tissues is suppressed in a significant way by integrated remediation systems, including the Bacillus subtilis coupled with organic amendments. Together with the naturally low movement of cadmium from roots to fruits, which ranges from 0.033 to 0.184, this helps ensure that Cd levels in the edible pericarp remain within safe regulatory limits [6,78].

The functional quality is characterised in the indigenous Bangladeshi cultivars such as Naga, Dhani and Kajini by an abundant amount of bioactive substances, which change with maturity levels. The vitamin C content of these cultivars varies between 1.67 and 8.45 mg g^−1^ FW, whereas the total phenolic content and total flavonoid content are 16.68–46.76 mg GAE/g and 2.80–8.53 mg QE/g, respectively. Antioxidant activity measured using FRAP and DPPH tests showed high values of 311.03 mM Fe(II) equivalents per 100 g dry weight and 329.52 µM Trolox equivalents per gram dry weight. Pungency has a high positive correlation with the developmental stage, with the pungency increasing as the fruit grows. Using GWAS, 4CL (Chr 1), BCKDH (Chr 1), and FatB (Chr 3) were crucial nodes that play a role in sustaining capsaicin and dihydrocapsaicin levels during stress [88]. These chemical components are strongly correlated (r = 0.80–0.97) with other bioactive profiles, indicating that the active selection of high-pungency characteristics (e.g., in the Naga variety) is also associated with the improvement of the antioxidant defence capacity of the fruit.

**Figure 5:**
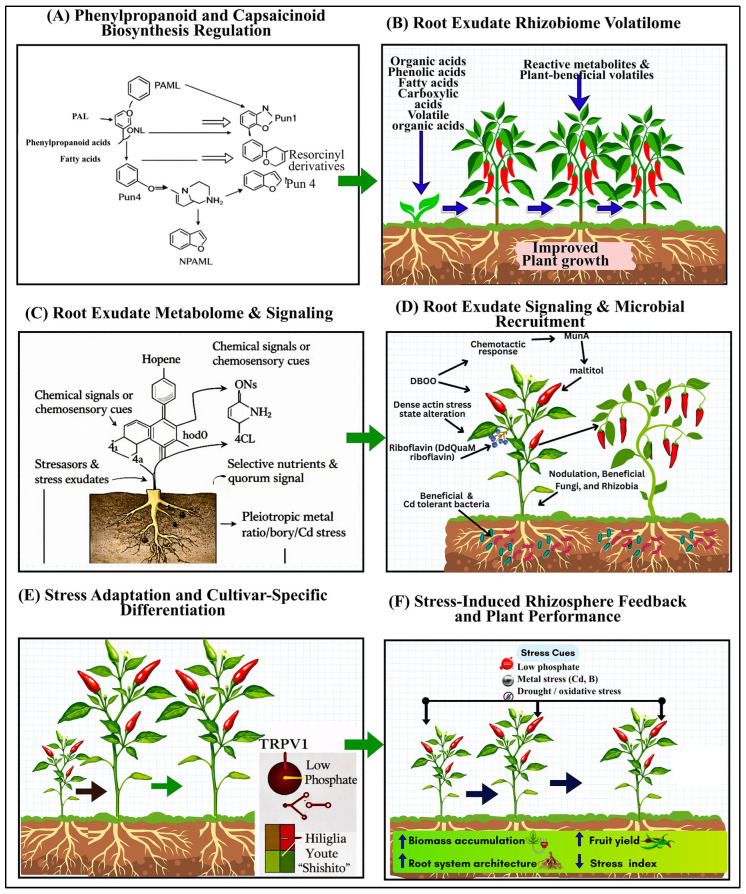
Integrated conceptual framework linking capsaicinoid biosynthesis, root exudation, and rhizosphere interactions under stress conditions in chilli pepper. Panels (**A**) depict regulation of phenylpropanoid and capsaicinoid biosynthetic pathways, (**B**,**C**) the composition and signaling roles of root-derived metabolites and volatile compounds, and (**D**) their association with microbial sensing and selective enrichment in the rhizosphere. Panels (**E**,**F**) illustrate cultivar-specific stress adaptation and rhizosphere feedback linked with plant growth, pungency expression, and performance under combined nutrient limitation, metal stress, and drought. The figure synthesis reported biochemical, physiological, and microbiome-level observations, highlighting coordinated patterns of plant–microbe interactions.

The pangenomic tools have helped in establishing cultivars that keep stable yields even under combined stresses such as heavy metal contamination and drought. Fruit traits show wide variation, with fruit weight ranging from 0.3 to 127.36 g and seed numbers varying from 0 to 295 per fruit [86]. Strong correlations between texture and physical traits (r = 0.70–0.99) indicate that when fruit texture remains stable, the overall external quality of the fruit is usually maintained as well. In Anaheim chilli and local varieties, some yield traits can be kept stable with the help of structured composites and beneficial rhizobacteria. These include fruit width, which ranges from 0.53 to 10.77 cm, and fruit length, which ranges from 0.97 to 19.23 cm. For example, using chitosan–maltodextrin at concentrations of 1000 and 2000 mg L^−1^ has been shown to increase total yield in dry regions. Genetic effects on traits like the firmness and consistency of fruits have large SNP effect sizes (e.g., −1352.69 to 286.86 firmness), indicating that these traits have complex oligogenic architectures, which can be manipulated to produce climate-resilient breeds 295 [86]. The combined process of soil Cd dynamics, cultivar-specific genetic signalling through the Pun1 and PAML loci, and its ultimate sequestration by specialised microbial consortia at the root level is visually modelled in Figure 5, as the route to optimised food safety and yield stability in two-stress conditions.

### 5.3. Ecosystem-Level Benefits

Capsaicinoid-modulated rhizobiomes engineering in chilli agroecosystems not only helps in ensuring the present health condition of the plants, but also a chain of environmental advantages that support the sustainability of the agroecosystems in the long term. These systems alleviate the negative impacts of intensive monoculture and climate-related stressors, including metal leaching and land degradation caused by the monsoon [89,90]. Longer crop rotation periods are linked with lower soil pH, but they are also associated with higher levels of available phosphorus and ammonium. Although lower soil pH can increase metal solubility, the buildup of organic matter and the activity of beneficial microbial communities can help bind these metals in the soil and reduce their availability. Leaching can be mitigated by using conservation agriculture methods, which are noted to increase water productivity by 18–66% compared to conventional methods [90]. These systems lead to the reduction of vertical migration of the contaminants into groundwater aquifers during high precipitation events as they enhance the soil carbon stabilization (12–93% increase in SOC).

The use of AMF and PGPR, and *Actinobacteria* in microbial consortia has benefits that are far more synergistic than that of the single inoculum. AMF develop symbiotic relationships with 93% of terrestrial plant families, increasing P, Fe, and Zn absorption besides improving the soil structure and water retention. *Bacillus*, *Pseudomonas*, and *Paenibacillus* genera produce phytohormones (e.g., AIA, GA3, ABA), enzymes (ACC deaminase), which lower ethylene levels and enhance root density. These taxa also produce organic acids (e.g., gluconic and citric acids) and volatile terpenoids that form protective layers against pathogens of the soil [8,85]. It has been reported that conservation agriculture-based management in a variety of Indian agroecosystems has grown crop productivity by 3.8–76.2% and energy-use efficiency by 8.9–40.2% [90]. When chilli monocultures are planted, the high pathogenic pressure in the first cultivation years can be reduced by long-term cultivation, as the beneficial microorganisms will eventually stabilize, thus reducing the symptoms of the disease [89].

Capsaicinoid-induced microbial recruitment, enhanced soil aggregation, and selective biofertilization guarantee the high nutraceutical value of produce and prevent soil erosion of the lithosphere. This hybridised approach reduces environmental pollution, the cause of more than 40% of the total land degradation, and protects the food chain against heavy metal pollution [8,90]. The combined use of microbial consortia and sustainable soil management practices leads to measurable improvements in soil quality, plant physiology, and stress resilience. Table 4 summarises key systems and practices, along with their quantitative effects on soil or microbial indicators and their functional outcomes under different stress conditions.

## 6. Synthesis, Knowledge Gaps, and Future Framework

The overall findings of recent genomic, metabolomic and field-scale studies create a multidimensional paradigm of Cd phytostabilisation in chilli pepper agroecosystems. This synthesis assesses the convergence of cultivar-specific secondary metabolism and rhizobiome engineering to hypothesise a data-based model of sustainable production in metal-polluted drought-stressed ecosystems. Studies using microarray-based approaches have provided valuable insights into how crop cultivars and their associated microbes evolve together. These investigations reveal that cultivar–microbe co-selection is a dynamic process, shaped by complex molecular interactions that vary across plant genotypes and environmental conditions. The development of a solid phytostabilisation model is based upon the joint co-selection of the host genotype and its associated microbial community. The quantitative data of various studies show that those types of chilli cultivars differ in their ability to retain metals in roots, depending on their economic growth strategy. In particular, plants with varieties that follow a Slow Economic Strategy show a stronger relationship between rhizosphere diversity and functional characteristics (root length and phosphorus) accumulation than plants with Fast Economic Strategy (FES) [60].

Network topological features can be used to determine the stability of these engineered rhizobiomes. It has been demonstrated in integrated analyses that Medium Economic Strategy networks are the most strongly connected and resilient networks. The capsaicinoid gradient is a selective filter in high-pungency cultivars such as Jolokia that can produce up to 391,000–1,000,000 Scoville Heat Units of capsaicin [16]. Although stochastic processes contribute to community assembly, quantitative modelling indicates that deterministic selection is particularly pronounced in fungal communities. As a result, high-heat cultivars tend to foster more stable and predictable microbial niches, favouring beneficial taxa such as *Streptomyces* and *Bacillus*.

In field conditions where soils are contaminated with multiple metals, combined exposure to Cd and Pb causes more severe physiological damage than either metal alone. Studies on *Cucurbita pepo*, which provides useful insights for solanaceous crops, show that this combined stress can double membrane damage, increasing electrolyte leakage by 103% and malondialdehyde levels by 90% [80]. Through the use of targeted microbial consortia, such as combinations of *Pseudomonas azotoformans*, *Serratia rubidaea*, *Paenibacillus pabuli*, and *Bacillus velezensis*, which lower Cd and Pb concentrations by approximately 89–91%. In real-world contamination scenarios, dried chilli peppers collected from the Guizhou mining region contained Cr (2.219 mg kg^−1^) and Pb (1.894 mg kg^−1^) at levels exceeding safety limits. However, the application of selected biochars significantly reduced estimated daily intake values, particularly for children, who represent the most vulnerable population group [18].

Although data on a fixed +1.5 °C temperature increase are limited, temperature-dependent vapour pressure deficit measurements from rehydration studies can serve as a useful proxy. At peak daytime temperatures (12:00–14:00 h), drought-stressed peppers exhibited a relative water content of 63.6% [84]. Additionally, stabilising yield under warm conditions was shown to require the genetic maintenance of the photosynthetic machinery. Specifically, overexpressing chloroplast-targeted chaperones such as AdDjSKI helps preserve the efficiency of photosystem II (PSII), highlighting the necessity of protecting the photosynthetic apparatus to maintain yield stability in warming climates [1].

In spite of such genomic data, there are still huge gaps in the translation of micro-scale observations to macro-scale ecosystem management. Capsicum volatilome is vast, and 2734 are identified [78]. Nevertheless, there is a lack of research on the dynamics of these compounds in soil in real time. Though in vitro experiments prove that volatile fatty acids of bacteria prevent germination of pathogens, the permanence of these messages in open-field soil, which is subjected to monsoon cycles and different matric potentials, is not quantified. Evidence shows that MHS vibrational signals have the capacity to upregulate the level of capsaicin 5.88-fold [37], yet the metabolic cost of this recruitment cue is not known in the long term.

The majority of the offered studies are based on a comparatively short period (12 h to 60 days). The data on monoculture (long-term monoculture, up to 10 years) indicate that nitrate reductase and alkaline phosphatase activities increase nonlinearly, whereas b-glucosidase activity does not change after 5 years [91]. In urgent terms, it is necessary to have data over the entire 10-year cycle to evaluate the ability of engineered microbial consortia to resist the gradual process of soil acidification (decrease in pH to about 5.3) observed in intensive chilli belts [6,89]. The practicability of utilising insect residual streams (IRS) or nano-selenium (Nano-Se) at the regional level needs additional confirmation. Recent evidence indicates that IRS supplementation (3% aboveground fresh biomass) enhances the growth of aboveground fresh biomass by 145% [51], and foliar Nano-Se (5 mg L^−1^) enhances capsaicin by 29.6% [40]. However, the existing literature has not established the economic feasibility of applying these treatments on a large scale across extensive spice-growing regions.

Based on the available evidence, it is recommended to implement a standardised protocol for applying these findings in both industrial and domestic cultivation. The next-generation breeding should lay emphasis on the Slow Economic Strategy characteristics since they have better rhizosphere incorporation. The screening has to use Scoville Heat Units as an effective indicator because the genotypes with high pungency, such as ‘Jolokia’ (391,000–1,000,000 SHU), exhibit the strongest antioxidant response [16]. Such varieties as X55 should be used because of their root retention, which has the lowest Cd migration coefficients to the fruit [32].

The most effective formulations identified consist of multi-strain consortia, including *Pseudomonas azotoformans*, *Serratia rubidaea*, and *Bacillus velezensis*. These consortia have shown an ability to cut down fruit Cd and Ni by more than 87% [79]. The inoculants are to be organised with the help of bone char carriers, which increase the solubility of phosphates in comparison to free bacterial suspensions by 26.9 times [78]. The regulatory systems need to consider the regional differences in metal speciation. In areas such as Guizhou, where Cr and Pb are over the safe limit in 33–89% of the samples [18]. The policy should require the use of 2% poultry manure biochar, which has been demonstrated to increase the number of fruits by 84.6% and protect against ozone and metal damage [82]. Conservation agriculture can also enhance soil organic carbon by 12–93%, effectively stabilising the lithosphere against vertical leaching [90].

Despite the promising outcomes reported for capsaicinoid-mediated rhizobiome engineering and Cd phytostabilisation, several critical knowledge gaps remain that may limit large-scale implementation. Most existing studies are short-term or pot-based, and robust quantitative evidence on the long-term stability of engineered rhizosphere communities under multi-year monocropping systems is still lacking. Moreover, while capsaicinoids and associated volatilomes are recognised as key drivers of microbial recruitment, their in situ dynamics, spatial influence, and persistence under field conditions remain poorly resolved. Soil-mediated feedback, including changes in pH, enzyme activity, and metal speciation, may further modify both microbial function and metal bioavailability, yet these interactions have not been systematically assessed. Addressing these limitations through long-term field trials, real-time volatilome monitoring, and region-specific assessments of metal chemistry will be essential to translate capsaicinoid-based strategies into durable, food-safe phytostabilisation systems.

The presented data prove that the process of capsicum phytostabilisation is regulated by a triplet interaction between high-pungency alleles (Pun1, Csy1), root retention proteins (HMA, NRAMP), and the functionality of microbial recruitment (Sphingobium, Bacillus). There are critical gaps in the literature that have not been resolved yet about the metabolic trade-offs of long-distance volatilome recruitment and the long-term stability of engineered inoculants under extreme pH changes. A hierarchical remediation strategy is recommended, beginning with the selection of SES-type cultivars (e.g., X55 or Naga), followed by the application of soil amendments such as 3% IRS or biochar. Finally, the approach is validated by analysing Cd and Pb partitioning in the fruit to ensure that levels remain below the safe consumption threshold of 0.05 mg kg^−1^.

## 7. Conclusions

Available evidence indicates that Cd phytostabilisation in chilli pepper agroecosystems emerges from the interaction between cultivar-specific secondary metabolism and rhizosphere microbial composition. High-pungency cultivars, characterised by elevated Scoville Heat Units, are consistently associated with specialised rhizobiomes that enhance root-level Cd sequestration and limit metal translocation to edible tissues. The application of multi-strain PGPR consortia and functional soil amendments, including biochar, further supports metal immobilization and yield stability under contaminated conditions. Despite these advances, major knowledge gaps remain. In particular, the long-term stability of engineered rhizobiomes under multi-year monocropping, changing soil chemistry, and fluctuating enzyme activity is poorly quantified. In addition, the in situ dynamics of the capsicum volatilome and its role in sustained microbial recruitment under field conditions remain largely unresolved. Addressing these gaps through long-term, region-specific field studies is essential for translating microbiome-assisted phytostabilisation into reliable and scalable management strategies for Cd-contaminated agroecosystems.

## Figures and Tables

**Table 1:** Regional patterns of Cd Phytoavailability and accumulation in Chilli Fruits.

Production Region	Phytoavailability/Fractionation	Fruit Cd (µg/g DW)	Risk, Compliance Status	Reference
Typical soils (China)	45.25% (EDTA-extractable)	0.007–0.345	High Cd treatments exceed safe limits	[6]
Shazand plain (Iran)	NR *	NR	PLI 71.17 (Severe Contamination)	[3]
Guizhou (China)—Total	Mining residues	0.045–0.194	Average 0.0627 µg/g	[18]
QDN region (Guizhou)	Mining residues	0.157 ± 0.037	Exceeds limit by 214%	[18]
LPS region (Guizhou)	Mining residues	0.115 ± 0.037	Exceeds limit by 130%	[18]
BJ region (Guizhou)	NR	0.064 ± 0.021	Exceeds limit by 28%	[18]
Serbian market (Fresh)	NR	<0.05	Generally safe compliance	[30]
Serbian yellow chilli	NR	>0.05	Identified as threshold exceedance	[30]
Greek green pepper	NR	>0.05	Identified as threshold exceedance	[30]
Balkan canned pepper	NR	<0.1 (ML)	Compliant with canned safety limits	[30]
Lanmuchang area (Mining)	Combined Tl-Cd stress	1.90 mg daily (Diet)	High Tl/Cd intake in residents	[25]
Indian powder (KT)	NR	NR	Guntur region agro-climatic notes	[31]
Alluvial soil (Mp)	Higher WHC/Rich NPK	NR	Lowers fruit proline/stress markers	[13]
Lateritic soil (Bp)	Low organic C	NR	Induces Pun12/Inhibits pungency	[13]
Lab spiked loamy soil	Homogeneous dispersion	NR	Decreased soluble sugar/proteins	[4]

* NR = Not Reported.

**Table 2:** Capsaicinoid content in representative chilli cultivars associated with beneficial rhizosphere functions.

Genotype	Parameter	Measured Concentration	Statistical Significance	Reference
Indian (Guntur type)	Total capsaicinoids	5.571 ± 0.139 g kg^−1^	*p* < 0.05	[49]
Indian (Guntur type)	Scoville Heat Units	85,909	*p* < 0.05	[49]
Jolokia	Scoville Heat Units	391,000–1,000,000	NR *	[16]
Jolokia	Ascorbic acid (AsA)	383 mg 1000 g^−1^ FW	*p* < 0.05	[16]
Khyati (KT)	Scoville Heat Units	16,526	NR	[31]
Khyati (KT)	Capsaicin	499.19 mg kg^−1^	NR	[31]
Shishito (SH/SH)	Total capsaicinoids	7958–10,658 µg g^−1^ DW	*p* < 0.001	[17]
Takanotsume (TK/TK)	Total capsaicinoids	17,817–18,245 µg g^−1^ DW	*p* < 0.001	[17]
Habanero	Pun1 allele	pun1^2^ (recessive, non-functional)	NR	[56]
*Capsicum annuum* L.	Phenylpropanoids	Lignin; phenolic acids (induced)	NR	[39]
*Capsicum longum*	Pathogen inhibitors	Robinin; rosmarinic acid (upregulated)	*p* < 0.05	[53]
*Capsicum longum*	Chemoattractants	Maltitol (increased)	*p* < 0.05	[53]
*Capsicum longum*	Stress signaling	Riboflavin (increased)	*p* < 0.05	[53]
KCa-4884	Water flux regulation	Aquaporins (12 genes upregulated)	NR	[36]
Healthy rhizosphere	Dominant genera	*Streptomyces*; *Bacillus*	*p* < 0.05	[51]
Cd-stressed chilli	Colonization efficiency	*Curtobacterium oceanosedimentum* (DG-20)	*p* < 0.05	[57]
Cd-stressed chilli	Tolerance level	18 mM Cd resistance	NR	[57]
*Pseudomonas* sp.	Cd-tolerance genes	*czcD*, *cadA*, *cadR*, *copC/D*	NR	[58]
Intercropped pepper	Recruitment marker	*Flavobacterium* (94% increase)	NR	[54]

* Statistical significance was not reported (NR) for some cultivars.

**Table 3:** Physiological and biochemical responses of *Capsicum* species to cadmium, drought, and hydric stress and their mitigation through microbial, physiological, and management-based strategies.

Physiological Parameter	Stress Condition	Control Value	Stress-Induced Response	Mitigated Outcome	Mitigation Strategy	Reference
Fruit Cd (µg/g)	Cd Stress	0.007	0.345	0.042 (88% reduction)	Consortia (Pa + Sr + Pp + Bv)	[79]
Leaf Cd (µg/g)	Cd Stress	NR *	High	88.5% reduction	PGPR Consortia	[79]
Root Cd retention	Cd Stress	NR	NR	40–55%	HMA1/Cell wall binding	[6]
Stomatal cond. (gs)	Cd Stress	Higher	Reduced	96% Increase	Consortia (Pa + Sr + Pp + Bv)	[79]
Photosynthetic rate	Cd Stress	Higher	Reduced	86% Increase	PGPR Consortia	[79]
RWC (%)	Drought (14d)	86%	51.36%	84.78% Recovery	Rehydration + Osmoprotectants	[7]
Electrolyte Leakage	Drought (14d)	19%	58.01%	25.01%	Rehydration recovery	[7]
Chlorophyll (µg/mL)	Drought (14d)	74.08	14.01	28.31 (100% Increase)	Rehydration (Day 15)	[7]
Proline (µg/g FW)	Drought (14d)	44.4	1489.27	NR	Drought response marker	[7]
Capsaicin content	Hydric Stress	Baseline	Variable	5.88-fold Increase	MHS Acoustic Emissions	[37]
Fresh biomass	Nutrient/Cd	NR	Reduced	145% Increase	3% Insect Residual Streams	[53]
Stem diameter	Cultivation	5.2 mm	NR	6.2 mm	3% IE Treatment	[53]
DTPA-extractable Cd	Soil Stress	CK	High	53.1% reduction	BBC-BS + Smooth Vetch	[78]
Fruit number	Ozone/Cd	NR	Reduced	84.6% Increase	2% Poultry manure biochar	[82]
Shoot length	Cd Stress	100%	Reduced	60% Increase	*C. oceanosedimentum* DG-20	[57]

* NR = Not Reported.

**Table 4:** Integrated soil, microbial, and management practices enhancing yield stability and stress resilience in chilli agroecosystems.

Management Practice	Biological Indicator	Magnitude of Effect	Functional Response	Stress Condition	Reference
Biochar Amendment	Poultry manure biochar (2%)	84.6% increase	Fruit number stabilisation	Tropospheric ozone	[82]
BBC-BS System	*Bacillus subtilis* (immobilised)	26.9% increase	Elevated phosphate solubilization	Cd-contaminated farmland	[78]
BBC-BS + SV	DTPA-extractable Cd	53.1% decrease	Enhanced Cd passivation	Cd-contaminated farmland	[78]
PGPR Consortia	Stomatal conductance (*g_s_*)	88–96% increase	Preserved photosynthesis	Heavy metals (Cd, Pb, Ni)	[79]
PGPR Consortia	Chlorophyll *a* content	125–129% increase	Biomass restoration	Heavy metals (Cd, Pb, Ni)	[79]
Insect Frass (IRS)	Fresh biomass	122.1–145.0% increase	Enhanced productivity	Nutrient scarcity	[53]
Conservation Agriculture	Soil organic carbon	12–93% increase	Carbon stabilization	Land degradation (SDGs)	[90]
Conservation Agriculture	Water productivity	18–66% increase	Improved irrigation efficiency	Land degradation (SDGs)	[90]
Monoculture (10 years)	Nitrate reductase activity	Nonlinear increase	Strengthened N cycling	Long-term chilli cropping	[89]
Monoculture (10 years)	Soil pH	R = −0.77 (negative correlation)	Progressive acidification	Long-term chilli cropping	[89]
Intercropping	*Flavobacterium* (rhizosphere)	94% increase	Biocontrol recruitment	Pepper + Chinese chive	[54]
Intercropping	Cross-host microbial migration	69.54% of root microbiota	Functional complementarity	Pepper + Chinese chive	[54]
Biocontrol	*Bacillus* sp. LBF-01	9.04-fold reduction	Disease suppression	*Fusarium oxysporum* wilt	[81]
Biocontrol	Seedling vigor	592.8% increase	Improved establishment	*Fusarium oxysporum* wilt	[81]
Biostimulant (GOE + BHs)	Malondialdehyde	49% decrease	Reduced membrane damage	Cd + Pb toxicity	[80]

## Data Availability

The data supporting the findings of this study are available from the corresponding author upon reasonable request.

## Abbreviations

The following abbreviations are used in this manuscript:

AMF	Arbuscular mycorrhizal fungi
CAT	Catalase
CBGs	Capsaicinoid biosynthesis genes
Cd	Cadmium
DEGs	Differentially expressed genes
EDI	Estimated daily intake
ERTF	Ethylene-responsive transcription factor
GPX	Glutathione peroxidase
GWAS	Genome-wide association studies
ISR	Inducing systemic resistance
MES	Medium economic strategy
Ni	Nickel
NR	Not reported
NRAMP	Natural resistance-associated macrophage proteins
OCRs	Open chromatin regions
PGPR	Plant growth-promoting rhizobacteria
PLI	Pollution load indices
POD	Peroxidase
Pun1	Pungency locus 1
Pun4	Pungency locus 4
QTLs	Quantitative trait loci
RWC	Relative water content
SES	Slow economic strategy
SHU	Scoville heat units
SNP	Single nucleotide polymorphism
SOD	Superoxide dismutase
T2T	Telomere-to-telomere
TFBS	Transcription factor binding motifs
TFv	Total flavonoids
Tl	Thallium
TPh	Total phenolics
VOCs	Volatile organic compounds
WHC	Water holding capacity
